# Clinical efficacy, safety, and subjective experience based on ePRO in HIV‐infected individuals administered Bictegravir/Emtricitabine/Tenofovir Alafenamide in southwest China

**DOI:** 10.1002/iid3.974

**Published:** 2023-08-28

**Authors:** Linghong Kong, Xiaoxin Xie, Yanhua Fu, Lin Gan, Xiaoyan Yang, Shujing Ma, Hai Long

**Affiliations:** ^1^ The Key Laboratory of Environmental Pollution Monitoring and Disease Control, Ministry of Education, School of Public Health Guizhou Medical University Guiyang Guizhou China; ^2^ Department of Infection Guiyang Public Health Clinical Center Guiyang Guizhou China

**Keywords:** adverse events, antiretroviral therapy, Bictegravir/Emtricitabine/Tenofovir Alafenamide, ePRO, HIV infection, virological inhibition

## Abstract

**Background:**

Prospective studies examining long‐term therapeutic outcomes of the Bictegravir/Emtricitabine/Tenofovir Alafenamide (BIC/FTC/TAF) regimen in human immunodeficiency virus (HIV) infection remain limited. This study assessed the actual efficacy and safety of BIC/FTC/TAF in HIV‐infected individuals in southwest China.

**Methods:**

This was a single‐center, prospective study enrolling ART‐naïve (*n* = 32) and ART‐experienced (*n* = 177) HIV‐infected patients administered BIC/FTC/TAF treatment between March 2022 and August 2022. The data were collected until February 28, 2023. Virological reactions and adverse events to the treatment were recorded, and patient subjective feelings in the form of Electronic Patient Reporting Outcome (ePRO) were collected. The primary endpoint was the rate of patients with HIV viral load <50 copies/mL at Week 24.

**Results:**

At Week 24, 87.5% and 95.5% of ART‐naïve and ART‐experienced HIV patients had a viral load <50 copies/mL, respectively. CD4 cell counts in ART‐naïve and ART‐experienced patients increased significantly by 163.5 cells/μL (*p* = .002) and 55.0 cells/μL (*p* = .022), respectively. By Week 24, no patients had discontinued the BIC/FTC/TAF treatment due to adverse events. Based on ePRO data, ART‐naïve and ART‐experienced patients at Week 24 had stable disease symptom burden, quality of life, and depression level after treatment with BIC/FTC/TAF.

**Conclusion:**

BIC/FTC/TAF reduces the viral load in ART‐naïve patients with high viral load as well as ART‐experienced patients with residual viremia. The patient's subjective experience was maintained stable after treatment with BIC/FTC/TAF. This study also revealed a very low incidence for BIC/FTC/TAF drug‐related side effects.

## Graphical abstract

This study suggests BIC+FTC+TAF is a safe option for achieving and maintaining virological suppression, both in treatment‐naïve patients with high viral load and treatment‐experienced patients with baseline residual viremia. Since there were almost no drug‐related adverse events with this regimen, and patients' subjective experiences of their own disease symptom burdens, quality of life, and depression levels based on ePRO after treatment with this regimen remained stable, it can be deduced that patient compliance can be ensured. In this study, significant decreases in ALT, AST, and GLU levels were firstly detected in treatment‐naïve and treatment‐experienced patients at Week 24. In the future, the sample size and follow‐up time should be increased to confirm these findings."

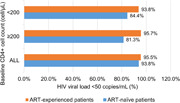

## 1 BACKGROUND

The effect of antiretroviral therapy (ART) in human immunodeficiency virus (HIV)‐infected cases largely determines patient prognosis. Patient compliance to ART determines treatment efficacy. To this end, there is an increasing international trend to apply compound single tablets with fixed doses to reduce the tablet burden on the patients, thereby ensuring patient compliance and improving the effectiveness of ART.^1^ Biktarvy Tablets is a three‐in‐one fixed‐dose compound single tablet drug, which comprises Bictegravir Sodium (50 mg), Emtricitabine (200 mg) and Tenofovir Alafenamide Fumarate (25 mg). It has been marketed in China since 2020 and was included in the 21st edition of the diagnostic and treatment guidelines at the end of 2021, becoming a new treatment of choice for HIV‐infected people. However, real‐world studies examining the clinical efficacy and safety of this regimen remain limited. Since the drug was launched in Guiyang Public Health Treatment Center, it has been used by some patients. This study aimed to provide a clinical experience for the selection of antivirals in newly diagnosed HIV‐infected patients by analyzing the data of patients administered Biktarvy Tablets.

Patient Reporting Outcome (PRO) is not interpreted by medical professionals, as a report directly from patients concerning their own health status and treatment effectiveness.^2^ PRO evaluation is usually based on a series of standardized scales, which are utilized as assessment tools comprising a clear conceptual framework and require multiple scientific validation steps before use. As the most direct and accurate feedback tool for evaluating drug efficacy, PRO is widely applied in clinical trials. Applying PRO data is a direct reflection of the patient‐centered clinical efficacy evaluation model. ePRO, as an electronic PRO data collection tool based on electronic operating systems, has the advantages of timely data filling and uploading, and convenient management. It is increasingly applied in clinical trials.^3^ However, in the clinical research on AIDS treatment in China, the application of ePRO is still in its infancy. This study used the HIV Symptom Index (HIV‐SI), the European Quality of Life Five Dimension Five Level Scale questionnaire (EQ‐5D‐5L), and the Patient Health Questionnaire‐9 (PHQ‐9) to assess the efficacy and safety of drugs, to collect patients' experiences and feelings, and to guide and adjust further clinical treatment.

## 2 METHODS

### 2.1 Study design and setup

This single‐center prospective cohort study recruited ART‐naïve (*n* = 32) and ART‐experienced (*n* = 177) HIV‐infected patients administered BIC (50 mg)/FTC (200 mg)/TAF (25 mg) from March 2022 to August 2022. The patients were followed up until February 28, 2023.

### 2.2 Ethics

The study followed the Declaration of Helsinki and was approved by the Ethics Committee of Guiyang Public Health Treatment Center (REC: 202226). All participating patients provided signed informed consent.

### 2.3 Inclusion criteria

Inclusion criteria were: (1) HIV infection; (2) ≥18 years of age; (3) treatment with BIC/FTC/TAF between March 2022 and August 2022, with available baseline and 24‐week CD4 counts and viral load data.

### 2.4 Exclusion criteria

Exclusion criteria were: (1) pregnancy; (2) loss to follow‐up or death; (3) nonavailability of data such as viral load, CD4 count, and blood biochemistry indexes at baseline and 24 ± 4 weeks; or (4) inability to complete the HIV‐SI, the EQ‐5D‐5L, and the PHQ‐9 at the visit point.

### 2.5 Variables and data sources

Routine outpatient follow‐up was scheduled for Weeks 4, 12, and 24. The doctors scheduled the visits based on each patient's personal situation. The routine care is as follows: once ART was started, viral load, CD4 count, blood routine indexes, urine routine parameters, blood biochemistry indexes, electrocardiogram, chest X‐ray, ultrasound, and other related parameters were obtained for each patient at the baseline visit, and the symptom management, health status, and sunshine index scale was completed by the patient. At Weeks 4 and 12, the patient underwent routine blood and urine tests and blood biochemical tests, and completed the symptom management and sunshine index scale. At Week 24, viral load, CD4 count, blood and urine routine parameters, and blood biochemistry indexes were determined, and the patient completed the HIV‐SI, the EQ‐5D‐5L, and the PHQ‐9. Patient‐provided data were extracted from case report forms, the hospital information system, and electronic scale systems. Body weight, immune response, and blood biochemistry indexes were evaluated at baseline and Week 24, respectively. The clinical efficacy of BIC/FTC/TAF was evaluated based on virological inhibition and CD4 count increase at Week 24. All patient PRO scales were filled out through the digital information platform “Fortunately, You,” which is based on WeChat, the most widely utilized social media platform in China. Each patient entered the scale and filled it by scanning the hospital's exclusive Quick Response Code. The PRO data were exported from the platform's background.

The effect of BIC/FTC/TAF treatment on the patient's subjective feelings was examined based on changes in the HIV‐SI, the EQ‐5D‐5L, and the PHQ‐9 scores. Safety was assessed throughout the study, recording adverse events, drug‐related adverse events, severe adverse events, BIC/FTC/TAF treatment discontinuation, and laboratory indicators. The primary endpoint was the proportion of patients with HIV viral load <50 copies/mL at Week 24. Secondary endpoints included changes in body weight, CD4 count, blood biochemistry indexes, and adverse events at Week 24, and changes in HIV‐SI, EQ‐5D‐5L, and PHQ‐9 scores. Blood lipid abnormality was defined as high total cholesterol (TC) ≥ 5.2 mmol/L, triglycerides (TG) ≥ 1.7 mmol/L, low high‐density lipoprotein cholesterol (HDL‐C) < 1.0 mmol/L), or low density lipoprotein cholesterol (LDL‐C) ≥ 3.4 mmol/L.^4^ Renal dysfunction was defined as creatinine clearance ≤80 mL/min. Abnormal liver function was defined as serum glutamic pyruvic transaminase ≥50 U/L or glutamic oxalic transaminase ≥45 U/L.

The HIV‐SI is a validated PRO tool used to assess the burdens of 20 common symptoms associated with HIV treatment or disease.^5^ This tool is considered the gold standard in contemporary HIV symptom research.^6^ Respondents were requested to describe their experiences with 20 symptoms over the past 4 weeks. The response options and scores were as follows: (0) “I don't have this symptom;” (1) “I have this symptom, it does not bother me;” (2) “I have this symptom, it concerns me a bit;” (3) “I have this symptom, it troubles me;” (4) “I have this symptom, which bothers me very much.” The 20 symptoms that constitute the HIV‐SI include fatigue/energy loss, insomnia, tension/anxiety, diarrhea, changes in body composition, feeling sad/depressed, abdominal distension/stomachache/flatulence, muscle soreness/joint pain, decreased sexual ability, poor memory, headache, hand/foot pain/numbness/tingling, skin problems/rash/itching, cough/difficulty breathing, fever/chills/sweating, dizziness, weight loss/weight gain, nausea/vomiting, alopecia, and loss of appetite/loss of food taste.

The EQ‐5D‐5L, developed by the European Organization for Quality of Life (Euro QoL Group), has two parts, including the health description system (EQ‐5D descriptive system) and the visual simulation scale (EQ‐VAS). The first part comprises five dimensions, including mobility, self‐care, daily activities, pain or discomfort, and anxiety or depression. Each dimension contains five options and scores: (1) no difficulty; (2) somewhat difficult; (3) moderately difficult; (4) severely difficult; (5) extremely difficult. The visual simulation scale scores between 0 (worst state of health) and 100 (complete state of health or the individual's best state of health). The respondents rate the individual's health on a given day. The Chinese version of the EQ‐5D‐5L scale has been evaluated for reliability and validity by domestic scholars.^7^

The PHQ‐9 is an internationally accepted self‐rating depression scale used for screening depression symptoms. It is simple and convenient, and has been shown to have good reliability and validity.^8^, ^9^ The latter scale contains 9 items, with 4 options each, from a score of 0 (no knowledge at all) and 3 (almost every day), for a maximum total score of 27 points. The higher the score, the higher the severity of depressive symptoms. In this study, the cut‐off value was set to 5, that is, ≥5 was considered to indicate depression.

### 2.6 Statistical analysis

Qualitative variables were reported as frequency distributions, while quantitative data were expressed as median and interquartile range (IQR) or mean and standard deviation (SD). The Kolmogorov–Smirnov test was used to assess the normality of numerical variables. The Student's *t* test was performed to compare normally distributed independent variables between baseline and Week 24, while the Mann–Whitney *U* test was used for nonnormally continuous variables. Statistical significance was set at *p* < .05. SPSS version 23.0 (IBM) was used for data analysis.

## 3 RESULTS

### 3.1 Patient characteristics

**Figure 1:**
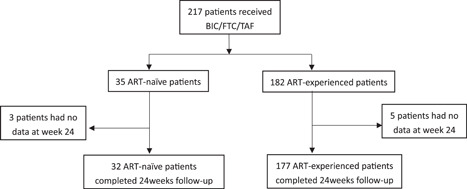
Study flowchart.

The patient selection process is summarized in Figure 1. A total of 217 patients were administered BIC/FTC/TAF for various reasons from January 2022 to August 2022. Two ART‐naïve patients and four ART‐experienced patients discontinued BIC/FTC/TAF due to economic difficulties. One ART‐naïve patient and one ART‐experienced patient transferred to other medical institutions. The study sample (*n* = 209) included 32 ART‐naïve and 177 ART‐experienced patients. The patient baseline characteristics are shown in Table 1.

**Table 1:** Baseline patient characteristics (*n* = 209).

Characteristic	ART‐naïve (*N* = 32)	ART‐experienced (*N* = 177)
Sex, *n* (%)		
Male	30 (93.6)	165 (93.2)
Female	2 (6.3)	12 (6.8)
Age in median (IQR), year	31.0 (27.5, 36.5)	36.0 (32.0, 43.0)
Age <45 years, *n* (%)	27 (84.4)	139 (78.5)
Transmission routes, *n* (%)		
MSM	22 (68.8)	115 (65.0)
Heterosexual intercourse	8 (25.0)	53 (29.9)
IDU	0 (0)	3 (1.7)
Unspecified	2 (6.3)	6 (3.4)
CD4 count in median (IQR), cell/μL	196.5 (46.5, 378.0)	430.0 (291.0, 572.0)
<200 cell/μL, *n* (%)	16 (50.0)	16 (9.0)
≥200 cell/μL, *n* (%)	16 (50.0)	161 (91.0)
HIV viral load in copies/mL, *n* (%)		
<50	0 (0)	158 (89.3)
50–499,999	22 (68.8)	19 (10.7)
≥500,000	10 (31.3)	0 (0)
Creatinine clearance ≤80 mL/min, *n* (%)	6 (18.8)	28 (15.8)
Comorbidities, *n* (%)		
Dyslipidemia	23 (71.9)	126 (71.2)
Cardiovascular disease	4 (12.5)	47 (26.6)
Hyperuricemia	5 (15.6)	71 (41.1)
Renal impairment	6 (18.8)	28 (15.8)
Hepatic impairment	8 (25.0)	39 (22.0)
AIDS‐related opportunistic infections, *n* (%)	13 (40.6)	69 (39.0)
Primary reasons for use of BIC/FTC/TAF, *n* (%)		
Dyslipidemia	4 (12.5)	35 (19.8)
Hepatic impairment	2 (6.3)	21 (11.9)
Renal impairment	4 (12.5)	14 (7.9)
Reduce drug burden	22 (68.8)	63 (35.6)
Side effects of previous ART	0 (0)	39 (22.0)
Others	0 (0)	5 (2.8)
Previous ART, *n* (%)		
2 NRTIs+NNRTIs	—	116 (65.5)
2 NRTIs+INSTIs	—	47 (26.6)
Others	—	14 (7.9)
Previous TDF‐based regimen	—	121 (68.4)
HIV‐SI score in median (IQR)	11.5 (4.0, 22.0)	16.5 (8.0, 26.0)
EQ‐5D‐5L score in median (IQR)	5.0 (5.0,6.0)	5.0 (5.0, 6.0)
EQ‐VAS score in median (IQR)	86.5 (50.0, 96.0)	85.0 (50.0, 94.5)
PHQ‐9 score, *n* (%)		
Depression	12 (37.5)	73 (41.2)

The ART‐naïve group mainly included men (93.6%), with a median age of 31.0 years (IQR, 27.5 years, 36.5 years), and 27 patients (84.4%) were younger than 45 years. In terms of infection route, 22 cases (68.8%) were MSM. In this patient group, 10 patients (31.3%) had a baseline HIV viral load >500,000 copies/mL, and 16 cases (50.0%) had a baseline CD4 count <200 cells/μL. The incidence of dyslipidemia was 71.9%. The main reason for choosing BIC/FTC/TAF was medication convenience (68.8%). Twelve patients (37.5%) had depression according to the PHQ‐9 score at baseline.

Most ART‐experienced patients were also male (93.2%), with a median age of 36.0 years (IQR, 32.0 years, 43.0 years), and 78.5% of patients were younger than 45 years. In terms of infection route, 115 cases (65.0%) were MSM. At baseline, 83 patients (89.2%) had virological inhibition (HIV viral load <50 copies/mL), and median CD4 count was 430.0 cells/μL (IQR, 291.0 cells/μL, 505.72 cells/μL). The main reasons for using BIC/FTC/TAF in this patient group included ease of administration (35.6%) and previous side effects of ART (22.0%). Before switching to BIC/FTC/TAF, 121 (68.4%) patients received tenofovir dipivoxil treatment (TDF), and 19 (10.7%) had detectable viral load levels (≥50 copies/mL). According to PHQ‐9 scores, 73 patients (41.2%) had depression.

### 3.2 Efficacy of BIC/FTC/TAF

**Figure 2:**
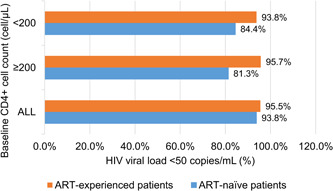
Analysis of patients with HIV viral load <50 copies/mL at Week 24.

At Week 24, the virological inhibition rate in the initial treatment group was 84.4% (27/32). The virological inhibition rates at Week 24 for baseline CD4 count <200 and ≥200 cells/μL were 81.3% and 93.8%, respectively (Figure 2). Of the 10 ART‐naïve patients with an HIV viral load ≥500,000 copies/mL, 7 (70.0%) had an HIV viral load <50 copies/mL following 24 weeks of treatment. Due to missing medication for 17, 21, and 25 days during the follow‐up period, HIV viral load in three ART‐naïve patients did not decrease to virological inhibition at 24 weeks, with 150, 261, and 118 copies/mL, respectively.

Of the 177 ART‐experienced patients, 169 (95.5%) had a viral load <50 copies/mL at Week 24. Baseline CD4 counts <200 and ≥200 cells/μL at Week 24 were 93.8% and 95.7% for ART‐experienced cases, respectively (Figure 2). Nineteen ART‐experienced patients had an HIV viral load ≥50 copies/mL at baseline, of whom 17 had concomitant diseases, and 89.5% (17/19) had an HIV viral load <50 copies/mL at Week 24. At Week 24, of the eight patients with an HIV viral load ≥50 copies/mL, three missed the treatment for 30, 40, and 45 days during the follow‐up period, respectively. Five patients developed persistent low‐level viremia while receiving ART, but there was no data on drug resistance.

### 3.3 Immune response and biological results

**Figure 3:**
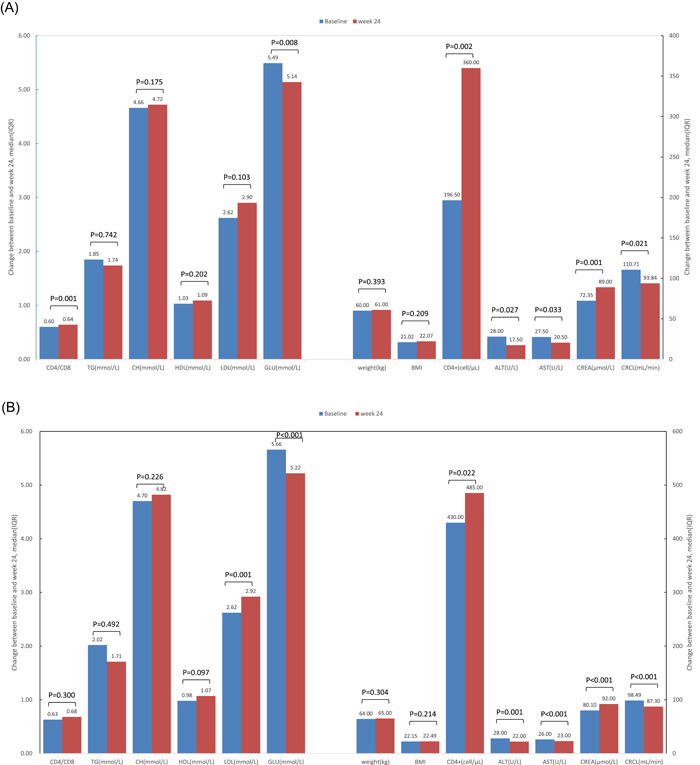
Changes in biochemical indicators between baseline and treatment Week 24. (A) Body weight, immune response, and biochemical indicators of ART‐naïve patients after treatment with the BIC/FTC/TAF regimen. (B) After treatment with the BIC/FTC/TAF regimen, body weight, immune response, and biochemical indicators in ART‐experienced patients were measured.

After 24 weeks of treatment with the BIC/FTC/TAF regimen, patient weight, immune response indexes, and biochemical indicators were obtained (Figure 3). The median CD4 count significantly increased by 163.5 cells/μL in 32 ART‐naïve patients (*p* = .002), while the CD4+/CD8+ ratio significantly increased by 0.135 (*p* = .001). ALT, AST, CRCL, and blood glucose were significantly decreased (*p* = .027, *p* = .033, *p* = .021 and *p* = .008, respectively), and CREA was significantly increased (*p* = .001). From baseline to Week 24, no significant changes were noted in body weight, BMI, CD8 count, TG, CH, HDL, and LDL (all *p* > .05, Figure 3A).

The median CD4 cell count significantly increased by 55.0 cells/μL in 177 ART‐experienced patients; (*p* = .022), ALT, AST, CRCL, and GLU significantly decreased (*p* = .001, *p* < .001, *p* < .001 and *p* < .001, respectively). LDL and CREA significantly increased (*p* = .001 and *p* < .001, respectively). There were no significant changes in body weight, BMI, CD4+/CD8+ ratio, TG, CH, and HDL (all *p* > .05, Figure 3B).

### 3.4 ePRO data

**Figure 4:**
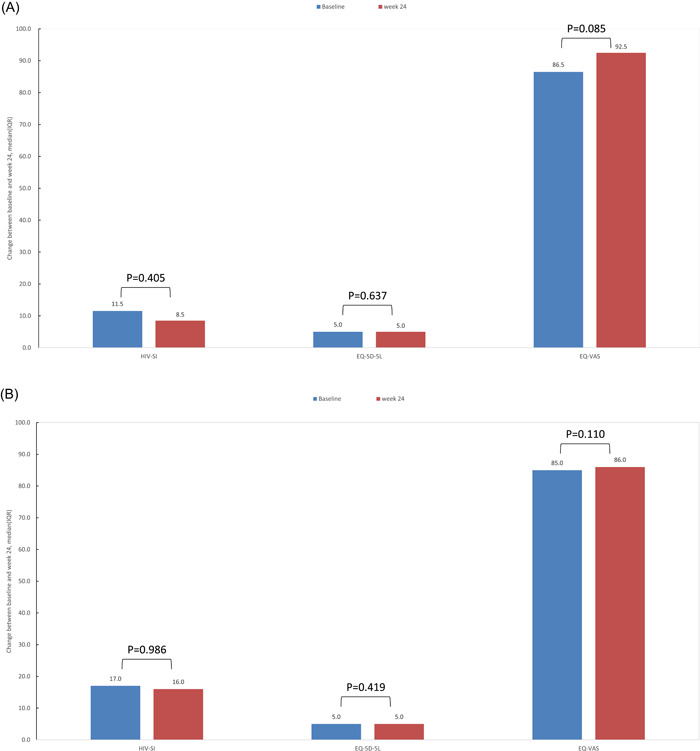
Changes in HIV Symptom Index (HIV‐SI), European Quality of Life Five Dimension Five Level Scale questionnaire (EQ‐5D‐5L) and Visual Simulation Scale (EQ‐VAS) scores between baseline and Week 24. (A) After treatment with the BIC/FTC/TAF regimen, ART‐naïve patients were assessed by the HIV Symptom Index (HIV‐SI), the European Quality of Life Five Dimension Five Level Scale questionnaire (EQ‐5D‐5L), and the Visual Simulation Scale (EQ‐VAS). (B) After treatment with the BIC/FTC/TAF regimen, HIV Symptom Index (HIV‐SI), European Quality of Life Five Dimension Five Level Scale questionnaire (EQ‐5D‐5L), and Visual Simulation Scale (EQ‐VAS) scores in ART‐experienced patients were evaluated.

After 24 weeks of treatment with the BIC/FTC/TAF regimen, changes in HIV‐SI, EQ‐5D‐5L, and EQ‐VAS scores were determined (Figure 4). Summary of changes in PHQ‐9 scores are summarized in Table 2. Among the 32 ART‐naïve patients, no significant changes were found in HIV‐SI, EQ‐5D‐5L, and EQ‐VAS scores (*p* = .405, *p* = .637 and *p* = .085, respectively). Among the 177 ART‐experienced patients, no significant changes were found in HIV‐SI, EQ‐5D‐5L, and Visual Analog Scale (EQ‐VAS) scores (*p* = .986, *p* = .419, and *p* = .110, respectively). The proportion of patients with depression in the initial treatment group showed no change, while the proportion of cases with depression in ART‐experienced cases increased slightly, with no statistically significant difference.

**Table 2:** Changes in Patient Health Questionnaire‐9 (PHQ‐9) scores between baseline and treatment Week 24.

Factor	Depression, *N* (%)	
	ART‐naïve (*n* = 32)	ART‐experienced (*n* = 177)
Baseline	12 (37.5)	73 (41.2)
Week 24	11 (34.4)	82 (46.3)
χ^2^	0.068	0.930
*p*	0.794	0.335

### 3.5 Adverse events

The estimated incidences of drug‐related adverse events in the ART‐naïve and ART‐experienced groups were 25.0% and 35.6%, respectively (Table 3).

**Table 3:** Adverse events related to BIC/FTC/TAF.

Adverse event	ART‐naïve, *N* (%)	ART‐experienced, *N* (%)
Drug‐related adverse event	8 (25.0)	63 (35.6)
Hepatic impairment	1 (3.1)	11 (6.2)
Renal impairment	1 (3.1)	35 (19.8)
Dyslipidemia	6 (18.8)	17 (9.6)
Treatment discontinuation due to adverse event	0 (0)	0 (0)
Serious adverse event	0 (0)	0 (0)

No adverse reactions resulted in discontinuation of BIC/FTC/TAF treatment by Week 24. There were also no serious adverse events that required patient hospitalization.

## 4 DISCUSSION

In a study of BIC/FTC/TAF treatment in southwestern China, high virus inhibition rates were reported in both ART‐naïve and ART‐experienced patients at Week 24. No patients discontinued BIC/FTC/TAF treatment due to adverse events. Six patients discontinued BIC/FTC/TAF due to economic difficulties. Two patients transferred to other medical institutions. Virological and immunological indicators are reliable for predicting disease progression and evaluating antiviral efficacy. These markers can also independently predict disease progression and patient survival in AIDS.^10^ A study of BIC/FTC/TAF demonstrated that the efficacy of this regimen is not inferior to that of a tenofovir‐based regimen in patients initially treated and receiving virological suppression from another regimen.^11^

In this study, three ART‐naïve and eight ART‐experienced patients had virological failure at Week 24; 10 (31.3%) of the 32 ART‐naïve patients had a baseline viral load >500,000 copies/mL, and 7 had a VL < 50 copies/mL at Week 24, consistent with previous findings.^12^, ^13^, ^14^, ^15^

After starting ART, CD4 count increased while CD8 count decreased.^16^ In this study, at Week 24 of BIC/FTC/TAF treatment, CD4 count in patients increased, indicating improved immune function after ART with BIC/FTC/TAF, corroborating previous studies.^12^, ^13^, ^14^, ^15^, ^17^

In patients with HIV and associated comorbidities, simplified medication regimens can reduce drug interactions and decrease the risk of renal dysfunction. In this study, 84.4% of patients with no previous ART and 78.5% of those with ART experience were younger than 45 years old. However, due to factors such as regional dietary habits, these patients still had high proportions of comorbidities, including cardiovascular diseases, cerebrovascular diseases, dyslipidemia, and impaired liver and kidney functions.

The main reason for choosing BIC/FTC/TAF was the low likelihood of drug interactions,^18^ indicating that this regimen is a good choice for patients with chronic noncommunicable comorbidities. Drug compliance is an important factor affecting ART efficacy, and poor compliance is the main risk factor limiting ART efficacy in HIV patients. If taken ART irregularly, the virus is not easily suppressed.^19^ Eleven patients experienced virological failure at 24 weeks, with six experiencing virological rebound due to missing medication.

In this study, blood creatinine levels significantly increased (*p* = .001 and *p* < .001, respectively), while creatinine clearance significantly decreased (*p* = .021 and *p* < .001, respectively) both in ART‐naïve and ART‐experienced patients at 24 weeks, but were within the normal ranges. This observation is consistent with previous findings.^20^

Dyslipidemia characterized by elevated LDL‐C levels is an important risk factor for atherosclerosis and cardiovascular disease.^4^ The prevalence of dyslipidemia in adult HIV/AIDS cases in China has reached 75.6%.^21^ In this cohort, a significant increase in LDL‐C (*p* = .001) was found after 24 weeks of BIC/FTC/TAF treatment in ART‐experienced patients, as well as a slight decrease in TG levels (*p* = .492) and a slight increase in HDL‐C (*p* = .097), which may not be clinically significant.

In addition, no significant changes in body weight and BMI were observed at 24 weeks. This finding contradicted Emond et al.^22^ but was consistent with another study.^23^ In Emond et al.'s study, patient weight changes were statistically significant only at 36 weeks. The reason for this discrepancy may be that the follow‐up period in this study was 24 weeks, and it may be necessary to extend the follow‐up period to assess long‐term changes.

We observed significant decreases in ALT, AST, and GLU levels in both ART‐naïve and ART‐experienced patients at Week 24 (*p* = .027 and *p* = .001, respectively; *p* = .033 and *p* < .001, respectively; *p* = .008 and *p* < .001, respectively), which may be the first time these phenomena are detected. In the future, we will expand the sample size and extend the follow‐up time to confirm these findings.

In this study, the ePRO system was used to subjectively evaluate patients based on three scales, and there were no significant changes in HIV‐SI, EQ‐5D‐5L, Visual Simulation Scale (EQ‐VAS), and PHQ‐9 scores in both ART‐naïve and ART‐experienced patients. This indicates that the subjective experiences of patients with their own disease symptom burdens, quality of life, and depression levels after treatment with the BIC/FTC/TAF regimen remained stable. In this study, there was also no discontinuation of the BIC/FTC/TAF regimen due to drug‐related adverse events occurring by Week 24, suggesting BIC/FTC/TAF is tolerable and safe, with no aggravation of adverse events based on the patient's subjective experience. This plays a very important role in improving patient compliance and ensuring adherence to long‐term treatment.

The main limitations of this study included its single‐center nature, small sample, and noncontrol design. In addition, restrictive exclusion criteria also limited information about the efficacy and safety of BIC/FTC/TAF in the excluded populations. Furthermore, patients were generally young and middle‐aged male, which may lead to limited applicability of these research results in the elderly or female population. Moreover, the follow‐up period of this study was only 24 weeks. In future studies, the follow‐up period may be extended to 48 or 96 weeks to observe long‐term efficacy, safety, and subjective patient experience for the BIC/FTC/TAF regimen.

## 5 CONCLUSION

This study suggests BIC/FTC/TAF is a safe option for achieving and maintaining virological suppression, both in ART‐naïve patients with high viral load and ART‐experienced patients with baseline residual viremia. Since there were almost no drug‐related adverse events with this regimen, and patients' subjective experiences of their own disease symptom burdens, quality of life, and depression levels based on ePRO after treatment with this regimen remained stable, it can be deduced that patient compliance can be ensured. In this study, significant decreases in ALT, AST, and GLU levels were first detected in ART‐naïve and ART‐experienced patients at Week 24. In the future, the sample size and follow‐up time should be increased to confirm these findings.

## AUTHOR CONTRIBUTIONS

**Linghong Kong**: Formal analysis; investigation; methodology; visualization; writing—original draft. **Xiaoxin Xie**: Conceptualization. **Yanhua Fu**: Methodology. **Lin Gan**: Conceptualization; methodology; project administration. **Xiaoyan Yang**: Data curation; supervision; visualization. **Shujing Ma**: Investigation. **Hai Long**: Conceptualization; project administration; resources.

## CONFLICT OF INTEREST STATEMENT

The authors declare no conflict of interest.

## ETHICS STATEMENT

The study followed the Declaration of Helsinki and was approved by the Ethics Committee of Guiyang Public Health Treatment Center (REC: 202226). All participating patients provided signed informed consent.

## Supporting information

Supporting information.

## Data Availability

The datasets used or analyzed during the current study available from the corresponding author on reasonable request. All data generated or analyzed during this study are included in this published article.
